# A Predictive Model of Pandemic Disaster Fear Caused by Coronavirus (COVID-19): Implications for Decision-Makers

**DOI:** 10.3390/ijerph19020652

**Published:** 2022-01-07

**Authors:** Vladimir M. Cvetković, Neda Nikolić, Adem Ocal, Jovana Martinović, Aleksandar Dragašević

**Affiliations:** 1 Faculty of Security Studies, University of Belgrade, Gospodara Vučića 50, 11040 Belgrade, Serbia; 2 Scientific-Professional Society for Disaster Risk Management, Dimitrija Tucovića 121, 11040 Belgrade, Serbia; jovanamartino@gmail.com; 3 International Institute for Disaster Research, Dimitrija Tucović 121, 11056 Belgrade, Serbia; 4 Faculty of Technical Sciences, University of Kragujevac, 32102 Cacak, Serbia; neda.nikolic@ftn.kg.ac.rs (N.N.); aleksandar.dragasevic@ftn.kg.ac.rs (A.D.); 5 Independent Researcher, Ankara 06500, Turkey; ocadem@gmail.com

**Keywords:** disaster, epidemic, pandemic, fear, COVID-19, predictive model, implications, decision-makers

## Abstract

This paper presents quantitative research results regarding a predictive model of pandemic disaster fear caused by the coronavirus disease (COVİD-19). The aim of this paper was to establish the level and impact of certain demographic and socioeconomic characteristics on pandemic disaster fear caused by the coronavirus (COVID-19). The research was conducted using a questionnaire that was provided and then collected online for 1226 respondents during May 2021. A closed, five-point Likert scale was used to create the structured questionnaire. The first section of the questionnaire included research questions about the participants’ socioeconomic and demographic characteristics, while the second section included issue questions about fear caused by COVID-19. The results of multivariate regression analyses showed the most important predictor for fear of COVID-19 to be gender, followed by age and education level. Furthermore, the results of *t*-tests showed statistically significant differences between men and women in terms of different aspects of pandemic disaster fear caused by the coronavirus disease. Our results have several significant public health implications. Women who were more educated and knowledgeable, married, and older, reported a greater fear of the outbreak at various levels. Decision-makers can use these findings to identify better strategic opportunities for pandemic disaster risk management.

## 1. Introduction

The coronavirus pandemic, which quickly crossed the borders of all countries, brought dramatic changes to people’s lives in various ways and affected all age groups. Although physical symptoms of the disease were a priority in the treatment of patients with coronavirus disease, the disease also caused significant mental problems [1], of which a significant increase in fear [2] associated with greater depression, stress and anxiety dominated [3,4,5]; these experiences were especially on the rise in the first months of the pandemic [6]. The possibility of accentuating already existing psychological influences due to new waves of coronavirus disease generates significant challenges and requirements for mental health professionals. This is evidenced by the reports of the World Health Organization (WHO), which express concern about the mental health and psycho-social consequences of the pandemic [7].

Sustained stressors associated with the pandemic, to which the entire world population is exposed and which, directly and indirectly, generate mental health problems, include social isolation, job loss, stress, and fear of infection or death of self or loved ones, as well as fear of an unknown, invisible enemy which persistently threatens them [8,9]. As a result, changes in activities, routines, and livelihoods have led to increased feelings of loneliness, depression, anxiety, insomnia, alcohol and drug use, self-harm, and suicidal behavior [7].

In parallel with the growing number of infected cases worldwide, neurocognitive symptoms caused by the pandemic have increased, which include delirium, acute and chronic impairment of attention and memory—related to hippocampal and cortical impairment, lack of learning in children and adults [10], and psychological illnesses, while healthy people with anxiety, panic and fear symptoms have also not remained immune [11]. Feng et al. [12] pointed out that individual variations in the internal organization of the fear circuit represent a neurofunctional marker that makes subjects vulnerable to experiencing high levels of fear during the COVİD-19 pandemic. The high degree of uncertainty associated with new pathogens has played a substantial role in the psychological impact of suspected or confirmed cases of coronavirus for individuals and healthcare professionals [13].

Coinciding with the spread of the pandemic, there has been an expansion of the infodemia regarding etiology, prevention, and disease outcomes. An essential concern in researching the structure of coronavirus disease fear is the sharing of emotional content on different networks and the creation of a particular emotional climate in society through this communication. In this regard, Pasquel-Lopez and Valerio-Urena [14] pointed out the importance of awareness of the impact of information on other people’s emotions. The results of a study conducted by Wheaton et al. [15] indicated greater susceptibility to emotional contagion during a pandemic in those more concerned about the spread of coronavirus disease, and experiencing higher depression, anxiety, and stress. In addition, in line with the results of previous studies, they identified media consumption as a predictor of coronavirus disease anxiety [15].

An analysis of a large number of tweets (3,308,476) indicated the wide prevalence and intensity of negative feelings, the percentage of which gradually increased after key events, and the importance of integrating social media for surveillance purposes in pandemic management as a preventive method to prevent emotional contagion by disseminating reliable information and cultivating trust [16]. At a time when rumors and large amounts of misinformation threatened psychological health, services such as the WhatsApp application adopted by the WHO and government agencies to inform the public about the coronavirus disease have contributed to resilience, the timely transmission of critical information, and, most importantly, the use of official WhatsApp channels which have emerged as a protective tool against psychological disorders, such as anxiety and depression [17]. Steinert [18] pointed to the potential for generating changes in social values through the sharing of emotional content online which leads to emotional contagion that could ease or aggravate the prevailing emotional climate, which can then affect the value structure of society.

The role of crisis communication during a global pandemic is crucial in combating various threats to mental health and minimizing and eliminating uncertainties and fears. Given the long-term consequences of inadequate communication, both individually and globally, a significant body of research has been devoted to examining the relationship between amounts of information related to coronavirus disease and mortality and mental health risk; a consensus has been reached on the minimal required media impact of newspaper organizations in the fight against the widespread coronavirus disease infodemia, while there has been shown to be inadequacy in communication practices during disasters [19].

The display of panic around coronavirus disease on social media was identified as the most important predictor of psychological disorders in a study conducted by Li and colleagues, which also assessed emotions and family relationships, finding that social support and emotional intelligence were negatively associated with psychological disorders. Furthermore, they found that emotional intelligence, but not social support, moderated the link between psychiatric problems and pandemic exposure [20].

Given the impact of the coronavirus disease on the central nervous system in the brain leading to long-term neurological problems [11], and the differential distribution of psychological symptoms due to the pandemic, one of the priority actions has involved paying more attention to communities known to be mentally more vulnerable during the previous phases of the pandemic [13]. Such groups have been shown to include health workers, women [21], young people, those who lost their jobs during the crisis, and people with chronic diseases, among whom the most severe symptoms of psychological trauma have been reported [22]. Both patients and healthcare professionals should be an integral part of mental health strategies during a pandemic. Some of these strategies have included the provision of counseling services, the application of awareness-based therapy and optimism interventions, and provision of telecommunication tools for communication between patients and families. Healthcare staff need to be provided with appropriate and flexible shift arrangements and interventions to strengthen morale [23]. Moreover, different care services should pay more attention, and incorporate interventions, to address the perceived feeling of loneliness [24]. Resilience mediates the link between anxiety and fear and strengthening resilience should be a priority in maintaining mental health, a key component of subjective wellbeing and physical health, especially in difficult times, such as global pandemics [25].

### Literature Review

Analyses of psychological trends during the pandemic have highlighted the most vulnerable categories of the population, including especially patients and health workers [26,27,28,29,30,31,32,33,34]. Schimmenti and co-workers [35] have pointed out that fears of the coronavirus disease during a pandemic were grouped into four interrelated psychological domains, representing the cognitive, behavioral, interpersonal, and physical characteristics of fear: (1) fear of bodies/fear for the body, (2) fear of significant others/fear for significant others, (3) fear of ignorance/fear of knowledge, and (4) fear of taking action/fear of inaction. Improving the regulation of one’s own emotions and assessment of the body, promoting responsibility, and facilitating acceptance are some of the methods proposed by the authors in their article to minimize the impact of fear [35].

Different factors, such as individual differences in personality (e.g., perceptions, emotions, and behavior), contribute to different vulnerabilities and impairments, as well as individuals’ resilience to these consequences [36]. While previous diagnoses of mental illness further exacerbate and complicate the mental ill-health of individuals during a pandemic, age, male gender, and physical health are protective factors against psychological stress, including fear and perceived risk of infection [37]. Adherence to hygiene rules by people with pre-existing mental illness was directly influenced by the fear of infecting themselves and others [38]. Achieving a high level of awareness, resilience, and optimism has been found to weaken the pre-existing relationship between fear and depression, stress, and anxiety [36].

Starting from the undeniable importance of physical activity in clinical psychology as a means of combating anxiety and depressive disorders, the question has been raised as to whether physical activity affects the impact of the fear stimulus associated with the pandemic on otherwise healthy adolescents [39]. Wright et al. [40] confirmed that physical activity in adolescents is a protective factor during a pandemic, which can significantly suppress the negative effects of fear on their mental health and overall wellbeing.

Intolerance of uncertainty is significantly related to the population’s mental health, and this relationship is mediated by rumors and fear of the coronavirus disease [41]. With respect to clinical levels of depression and anxiety in adults, neuroticism, coronaphobia, and hypochondria stand out as dominant fear factors that predict pandemic-related psychopathology [42]. Neuroticism, accompanied by emotional reactivity and scarce resources for stress management when present to a significant extent, plays a central role in coping with a pandemic [43]. Some studies have demonstrated a link between the frequency of feelings of boredom in people and high neuroticism. These findings indicate that the relationship between neuroticism and boredom is associated with the influence of negative fantasies, and suggest that this could explain why boredom and fear are linked. Finally, it was found that a factor contributing to reducing the link between fear and boredom is a high perception of control over time [43].

A study conducted in Romania, which included 1340 adult respondents during the pandemic itself, confirmed the existential issues raised by the coronavirus disease pandemic. It identified an increase in feelings of loneliness, obsession with death, and preoccupation with God during quarantine, which also predicted fear of the coronavirus [44].

Increased exposure of health workers to the risk of various mental problems during a pandemic has contributed to the development of stress, depression, and anxiety, especially among those health workers who worked in the COVID-19 areas of hospitals, who reported a higher level of psychological problems [13]. Chow et al. [23] found similar psychological symptoms caused by a pandemic between patients and health care staff, including anxiety, fear, and depression. In patients, quarantine, life-threatening perceptions, and health status uncertainty may contribute to these psychological problems [23]. A higher prevalence of mental health problems has been identified in patients suspected of having coronavirus disease in the 41 to 50 age group with respiratory tract symptoms and for those who were quarantined at home [13]. In the case of health care staff, quarantine, together with the heavy workload of their work, fear of infection of self or a family member, witnessing poor patient conditions, and the requirement to wear protective equipment, were significant factors predicting anxiety [23].

A differential distribution of psychological consequences was found among hospital health workers. Barua et al. [45] found that 38.4% of physicians surveyed in their study had depression, 36.5% anxiety, 31.9% fear of coronavirus disease, and 18.6% insomnia, with inadequate resources in the workplace identified as the most significant predictor of all the psychological problems. By examining a large sample of the international community, Harper et al. [46] confirmed the above observations, highlighting fear as a predictor and generator of positive changes in human behavior (e.g., social distancing, improved hand hygiene, etc.) during pandemics. In a study conducted by Dahmen et al. [38], 68.6% of respondents reported that they were rarely or sometimes afraid, while 17.9% of respondents expressed a permanent fear of coronavirus disease infection or severe disease. Respondents who were affected by fear intended to adhere more frequently to the rules of hygiene and handwashing than those who were not afraid. Furthermore, the findings of the study indicated that the participants perceived seeking medical help as a risk, regardless of any mental or physical health changes [38].

Fear also had a profound psychological effect on smokers who, due to the presence of increased levels of fear, experienced increased motivation to quit smoking (which was predicted by the existence of medical comorbidities, but not increased age), and reduced smoking, suggesting the suitability of pandemic conditions for providing smoking cessation interventions [47]. However, the positive side of fear has been shown to be that vulnerability, and perceived risk of coronavirus disease, are reflected in their impact on increased engagement in preventive behavior during a pandemic [48].

Public fear increased substantially in parallel with the escalation of the pandemic over an observed 22-day period [49]; its corresponding decrease was recorded after the peak in April 2020 [50]. Significant findings of a study conducted by Sadiković et al. [51] indicated a decrease in the examined emotional states caused by the pandemic (e.g., worry, fear, boredom, and anger) over time, with the most significant decreases recorded for worry and then fear and boredom. According to the findings of their study, significant predictors of people’s emotional reactions included personality dimensions and specific behaviors in a pandemic. Their findings indicated significant associations between arousal of the activation system with worry, fear, boredom, and the fight against boredom and anger, and of the behavioral inhibition system with anger [51].

Three days after the WHO declared a coronavirus disease pandemic on 11 March 2020, Mertens et al. [52] conducted an online study on factors relating to psychological vulnerability, media exposure, and personal relevance of communications. They identified four dominant predictors of coronavirus disease fear: health anxiety, regular media use, social media use, and risks to loved ones. They also identified 16 different topics that generated concern among the population, of which the most significant were the health of loved ones, economic consequences, and the overload of health systems [52]. Increased exposure to the disease was positively associated with fear and concern, according to the findings of some studies [51]. Lin et al. [53] pointed to significant direct and indirect links between the problematic use of social media and psychological stress and insomnia, with fear of coronavirus disease being identified as an indirect effect for both. In addition to anxiety traits and the use of media as a predictor of fear, increased fear was also predicted by region [50]; in the US, fear was highest in regions with the most reported cases of coronavirus disease [21]. Lippold et al. [49] pointed out that the strongest predictors of increased levels of fear were unstable personality and neuroticism, as well as education, gender, and extent of risk. A detailed longitudinal analysis of a large sample of German citizens identified another significant predictor of fear—political orientation, with greater fear of coronavirus disease observed in conservatives compared to liberals. However, with growing perception of the threat of the virus, the influence of this variable disappeared. In contrast, personality remained a stable predictor [49].

The relationship between education and various psychological problems associated with coronavirus disease has been extensively discussed in the literature [54,55,56,57,58,59,60,61,62]. The most common types of fear that teachers and students may face during a pandemic are fear of family closure, fear of educational failure, and fear of losing social relationships [63]. In addition, as coronavirus disease affects the education of the future workforce, concerns for impact on future careers have been highlighted [64].

Differences in perception, as well as the fear of coronavirus disease, among different age groups, have been the subject of a significant number of theoretical and empirical studies [65,66,67], with a consensus that elderly members of the population are one of the most vulnerable risk groups in society [68].

Among older adults in Bangladesh, anxiety and feeling overwhelmed about coronavirus disease, as well as feelings of isolation from others and diagnoses of coronavirus disease for a family member or close friend, significantly predicted higher likelihood and degree of fear, while obtaining coronavirus-related information from health workers predicted a lower level of fear [69]. Similarly, Giordani et al. [70] found a positive association between diagnosis or death from coronavirus disease of relatives of respondents, and belonging to a high-risk group, with fear. The findings were corroborated by a study conducted in the Israeli population, which revealed a positive association between gender, socio-demographic status, chronic illness, belonging to a risk group, and the death of a family member from coronavirus disease, with fear of infection for themselves [71]. Mete et al. [72], examining the impact of the coronavirus disease pandemic on the fragility of the elderly, found that the majority (72.4%) were unable to receive routine health examinations, while during social isolation, chronic ailments increased by 39.3%, with fear of coronavirus disease infection, among other factors (e.g., present disease, delays in health examinations, etc.), significantly influencing increase in the fragility of the elderly [72]. During the pandemic, among dentists in Pakistan, widespread concern and fear of coronavirus disease infection were more prevalent in women and young and older dentists [73].

Life satisfaction is another significant predictor of stress and fear of coronavirus disease. For example, Dymecka et al. [74] found a positive correlation between fear of coronavirus disease and perceived stress, and a negative association with life satisfaction. The associations between fear of coronavirus disease and psychological stress and life satisfaction was also evidenced by the findings of a study conducted by Satici et al. [75]. Their study also highlighted the strong psychometric properties of the Turkish version of the coronavirus disease fear scale, which creates opportunities for further research into the various psychological effects of coronavirus disease in Turkey [75]. In a second study, Dymecka et al. [76] found that a sense of coherence mediates the relationship between stress and life satisfaction and that the relationship between stress and a sense of coherence is mitigated by fear of coronavirus disease [76]. In addition, Labrague and de Los Santos [77] pointed to the importance of job satisfaction in explaining the structure of coronavirus disease fear and that predictors of coronavirus disease fear include roles at work and coronavirus disease-related training. Fear was linked to lower work satisfaction, greater psychological stress, and increased organizational and professional intent to change [77].

The aim of this paper was to establish the level and impact of certain demographic and socioeconomic characteristics on pandemic disaster fear caused by the coronavirus (COVID-19). This paper posits the general hypothesis that knowledge predicts variables associated with disaster fear caused by the coronavirus disease (COVID-19). This research addresses research gaps in the field of disaster studies, as follows:

(a) disaster fear caused by the coronavirus disease has not been studied at an appropriate level; there is still a lack of reliable epidemiological data (COVID-19) on the psychological consequences of illness,
(b) insufficient examination of the influences of various factors on people’s disaster fear caused by the coronavirus disease, which is needed to improve disaster risk communication strategies, the quality of psycho-social support, etc.

## 2. Materials and Methods

**Figure 1:**
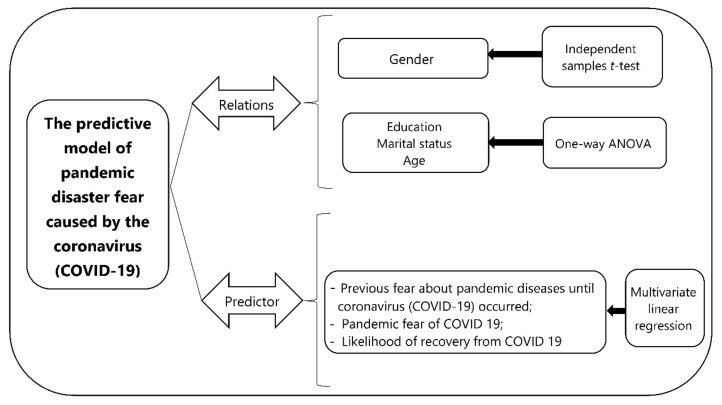
Research design.

This study using a quantitative approach evaluated a predictive model of pandemic disaster fear caused by the coronavirus disease (COVİD-19) (Figure 1). The research was conducted using a questionnaire that was administered online to 1226 people in May 2021.

### 2.1. Study Area

**Figure 2:**
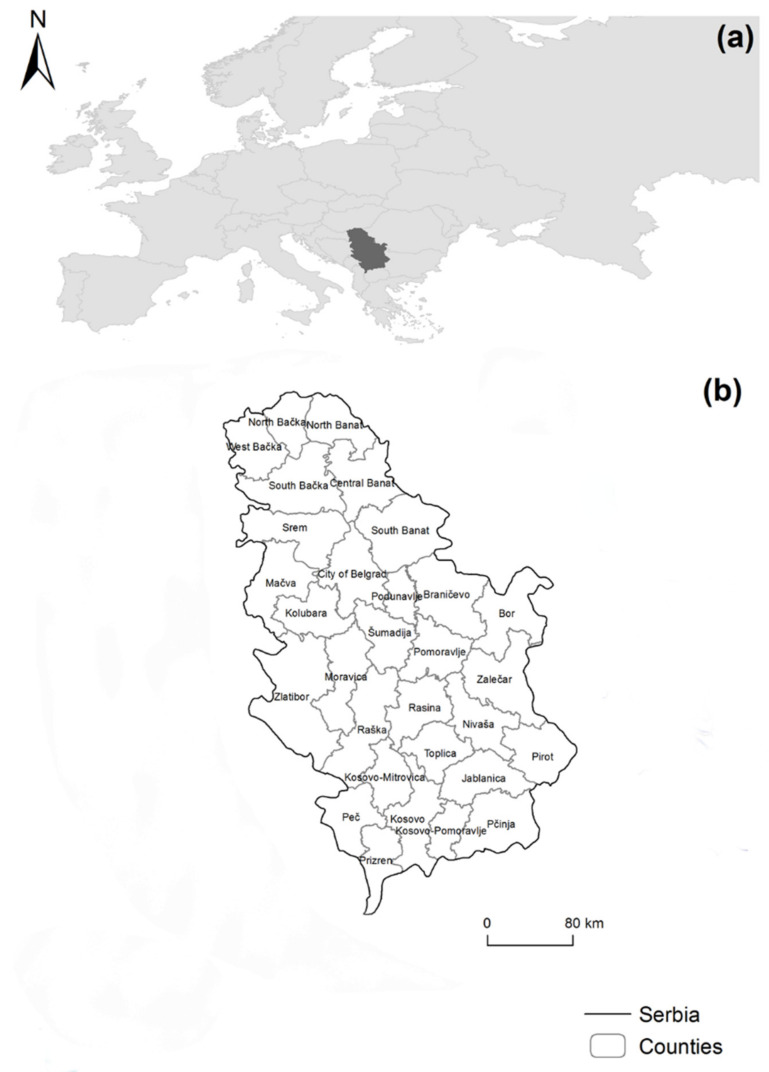
Study area. Location of Serbia (**a**); counties of Serbia (**b**).

The Republic of Serbia, which spans an area of 88.499 km^2^, is situated at the crossroads of central and south-eastern Europe in the Southern Pannonian Plain and the central Balkans. It is bordered on the north by Hungary, the northeast by Romania, the southeast by Bulgaria, the south by North Macedonia, the southwest by Montenegro, and the west by Croatia and Bosnia and Herzegovina (Figure 2). In addition, the disputed area of Kosovo, located in the country’s south-southwestern corner, shares a border with Albania. According to the 2011 census, the Republic of Serbia had a population of 7,186,862 people, with 3,499,176 men and 3,687,686 women [78,79]. 

Various epidemics have impacted the Republic of Serbia’s territory including plague (in the years 1348, 1362, 1428, 1430, and 1438), typhus (in the years 1914–1918), a smallpox epidemic in Yugoslavia in 1972, tularemia outbreaks in the 1990s, and the COVID-19 pandemic (in the years 2019–2021) [78]. After the Balkan wars, Serbia was completely unprepared, with respect to disease susceptibility and prevention, for the attack by the Austro-Hungarian army at the end of July 1914. The head of the Allied British Mission, the Colonel, Doctor Hunter, wrote in 1919: “The Serbian epidemic was thus the most sudden in origin, most rapid in rise, greatest in extent, and most quickly arrested of any epidemic of a like-kind in history. The curve of its origin, rise and decline, differs in character from that of any previous epidemic”, referring to the Irish (1847), English (1863–1865) and Serbian (1915) epidemics as the three great typhus epidemics [80]. Due to “the brilliant medical proposals for prevention” (one of these being Hunter’s recommendations that resulted in the famous “Serbian Barrel”), “government competence and the citizens’ responsibility in implementing them”, the epidemic was stopped [81]. Although assistance was necessary, especially at first, medical history research concludes that: “Serbian medicine has contributed scientifically to the medicine of the world in the field of combating and preventing freckled typhus by designing and applying the warm dry air chamber” [82]. The World Health Organization (WHO) had developed a *Variola major* eradication plan in 1967, and 10 years later the last case of smallpox was registered in Somalia. However, “the last large outbreak in Europe occurred in 1972 in Yugoslavia” with 175 cases and 35 deaths [83]. A pilgrim visited Mecca and Medina (Saudi Arabia) and returned home to Danjane village near Djakovica (Kosovo) by bus through Iraq when there were more smallpox cases. Before the eradication took place, the last case of smallpox in Serbia was registered in 1930, with the last lethal outcome in 1926 [81].

In Serbia, the first case of COVID-19 was reported on 6 March 2020, and was discovered as a consequence of many ill Serbian individuals coming home from nations where the pandemic had already started. When the pandemic first broke out in Serbia, the first action was to restrict all border crossings with neighboring countries, as had been done in other European countries. Citizens who obtained a self-isolation order or an epidemiologist’s order due to COVID-19 concerns or suspicions of contact with a sick person were required to stay at home for 14 or 28 days [84]. According to official data (updated on 12 September 2021) of the Institute of Public Health of Serbia, Dr Milan Jovanović Batut of the WHO stated that the total numbers infected were 1,270,407, hospitalized 3511, the number of people on respirators 197, the total number of deaths 12,075, mortality rate 0.95% and the total number tested 6,941,684 [85]. Even though history records a number of significant epidemics, researchers have discovered evidence of the Serbian public’s lack of understanding and readiness for epidemics [86]. COVID-19 hospitals and quarantine locations across the country treat patients with COVID-19 symptoms. If the test results are negative twice in a row, the patient is released. A prohibition of movement was introduced for all citizens from 5 pm to 5 am every day, which was later extended on weekends from Saturday at 3 pm (even 1 pm for several weeks) until Monday at 5 am. An exception was Easter weekend, during which the prohibition started on Friday at 5 pm and ended on Tuesday at 5 am. Starting from the April 21st, this category of citizens was granted the right to leave their household three times a week, on Tuesdays, Fridays and Sundays, between 6 pm and 1 am, for a maximum of half an hour and at a maximum of six hundred meters from the place of residence. In addition, the prohibition of movement for all citizens was changed; it started at 6 pm and lasted until 5 am the next day. The prohibition on gathering indoors first applied to more than 100 people, then applied to more than 50 people, with a maximum of two people at a distance between persons of at least two meters while on an area of four square meters. The government closed cafes and restaurants, casinos, bookmakers, casinos, hairdressers and beauty salons, car washes, all markets, both outdoors and indoors etc., and all facilities and outlets within shopping malls except supermarkets, grocery stores and pharmacies.

### 2.2. Socioeconomic and Demographic Characteristics

A total of 1357 people agreed to take part in the study, and 93 refused. Of the total number of respondents who agreed to participate in the survey, 1226 respondents completed the survey questionnaire, 131 respondents did not (the completion rate was 90.34%). The sample was 47.3% male and 52.6% female (population mean in Serbia is women 51.3% and men 48.7% [87]) with an average age of 30 years (mean population age in Serbia is men 41.2 and women 43.9). Of the participants, 53.02% were between 18 and 30 years of age, 31% were between 31 and 45 years, 15.9% were between 46 and 64 years. Married persons accounted for 41.8% of the study, while 40% were single. Of the respondents, 46.49% had one child. Those participants who considered themselves as having good COVID-19 knowledge comprised 38.3% (Table 1).

### 2.3. Questionnaire Design

In preparing the questionnaire for this study, several published survey approaches were consulted [48,68,88,89,90,91,92] and items were adapted to the properties of the Serbian demographic and socioeconomic status (Appendix A). In March 2021, a pilot test of the questionnaire was conducted in Belgrade (Serbia) with 45 participants to assess the questionnaire’s validity and reliability. Closed, five-point Likert scale questions (1 = strongly disagree to 5 = strongly agree) were used to create the structured questionnaire. The first section of the questionnaire included research questions about the participants’ socioeconomic and demographic characteristics, while the second section included issue questions about fear caused by COVID-19. Our quantitative analysis was consistent with the Helsinki Declaration [93], which established guidelines for socio-medical research involving human participants. The participants were obtained using a convenience sampling approach, and an initial invitation to participate in an online questionnaire was posted on social media. The study area covered all of Serbia. The research protocol was approved by the committees of the Institutional Review Board of the Scientific-Professional Society for Disaster Risk Management and the International Institute for Disaster Research (protocol code 003/2021, 10 September 2021).

### 2.4. Analyses

Descriptive statistics were obtained for the participants’ socioeconomic and demographic characteristics in this study. *t*-test [94], one-way ANOVA [95], and multivariate linear regression [96,97] were used to examine the relationships between the predictors and disaster fear caused by the coronavirus disease (COVID-19). The preliminary analysis of the homogeneity of variance (test of homogeneity of variances) showed a violation of the assumption of homogenous variance, so the results of two tests resistant to the violation of the assumption, Welch’s *t*-test and the Brown–Forsythe test, were used. All tests were two-tailed, with a significance level of *p* < 0.05. Statistical analysis was performed using SPSS Statistics (IBM SPSS Statistics, Version 26, New York, NY, USA). The internal consistency of the Likert scale, Reasons of Fear Citizens Subscale (15 items), was good with a Cronbach’s alpha of 0.84, while for the Information Sources of Citizens to Receive Information about Coronavirus Disease (COVID-19) subscale (9 items), Cronbach’s alpha was 0.81. We performed regression analyses with the five independent variables (gender, age, marital status, education level, children and knowledge of coronavirus) to examine the factors associated with the overall scale (Table 2). We tested the central hypothesis that knowledge predicts disaster fear caused by the coronavirus disease (COVID-19). A multivariate regression analysis was used, identifying the extent to which total scores of the primary dependent variables (e.g., previous fear, fear about COVID-19, recovery of yourself, information sources subscale) were associated with five demographic and socioeconomic variables: gender, age, marital status, education level, and knowledge. Previous analyses reviewing the residual scattering diagram [97] showed that the assumptions of normality (normal probability plot P-P and scatterplot), linearity, multicollinearity (*r* = 0.9), and homogeneity of variance had not been violated.

## 3. Results

The results were split into three groups based on the methodological framework and research design:

1. The predictors of fear scores related to fear caused by coronavirus disease (COVID-19);
2. Descriptive statistics and the relations between the variables and fear caused by coronavirus disease (COVID-19);
3. Gender perspectives about fear caused by coronavirus disease (COVID-19).

### 3.1. The Predictors of Fear Scores Related to Fear Caused by the Coronavirus Disease (COVID-19)

The multivariate regression analyses showed that the most important predictor for previous fear about pandemic diseases before coronavirus disease (COVID-19) occurred was gender (*β* = −0.119), explaining 1.2% of the variance in the score, followed by marital status (*β* = −0.085, 0.6%). The remaining variables did not have significant effects on previous fear. The model (*R^2^* = 0.021, *Adj. R^2^* = 0.016, *F* = 4.40, *t* = 25.31, *p* = 0.000) with all mentioned independent variables explained 2.1% of the variance of fear about pandemic diseases before coronavirus disease (COVID-19) occurred.

The results of the multivariate regressions showed that the most important predictor for fear of COVID-19 was gender (*β* = −0.099), which explained 0.8% of the variance in score; gender was followed by age (*β* = −0.098, 0.5%) and education level (*β* = −0.071, 0.49%). The remaining variables did not have significant effects on fear. This model (*R^2^* = 0.023, *Adj. R^2^* = 0.018, *F* = 4.79, *t* = 27.99, *p* = 0.000) with all mentioned independent variables explained 2.3% of the variance of fear about pandemic diseases before coronavirus disease (COVID-19) occurred (Table 2 and Figure 2).

**Figure 3:**
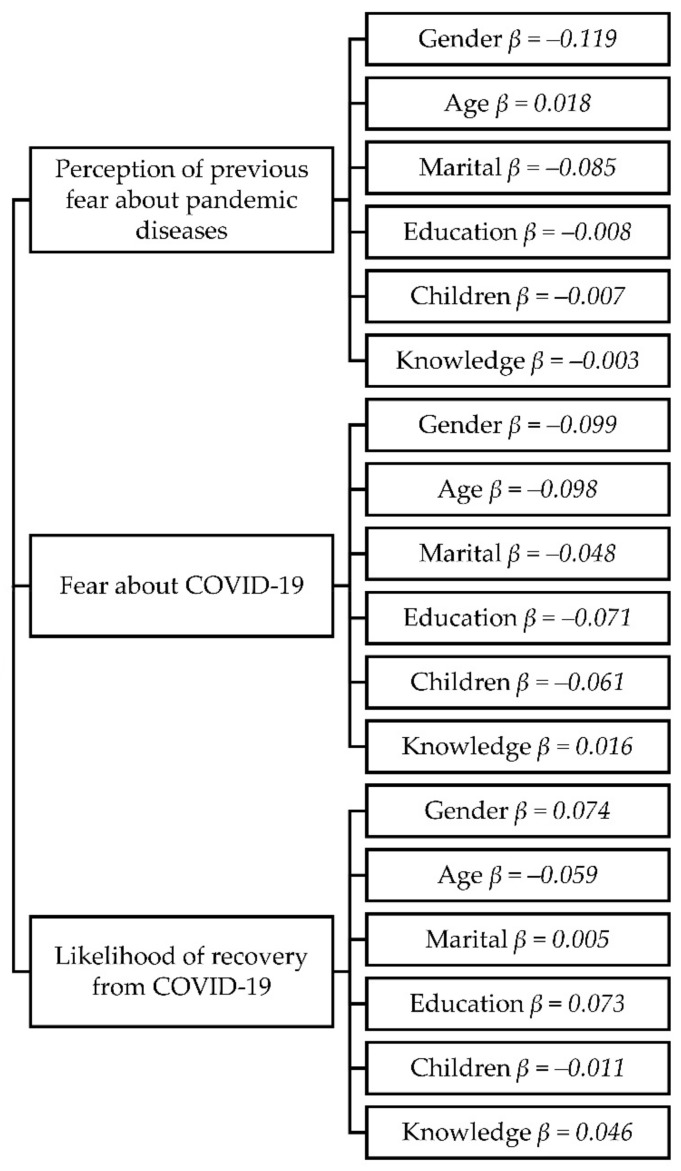
The predictors of coronavirus disease fear.

The results of the multivariate regressions showed that the most important predictor for recovery opinion from pandemic COVID-19 was gender (*β* = 0.074), explaining 0.54% of the variance in the score, followed by education (*β* = 0.073, 0.51%). The remaining variables did not have significant effects on recovery opinion. This model (*R^2^* = 0.017, *Adj. R^2^* = 0.012, *F* = 4.79, *t* = 3.43, *p = 0.002),* with all mentioned independent variables, explained 1.7% of the variance of recovery opinion from pandemic diseases before coronavirus disease (COVID-19) occurred (Table 2 and Figure 3).

### 3.2. Descriptive Statistics: The Relations between the Variables and Fear Caused by COVID-19

**Figure 4:**
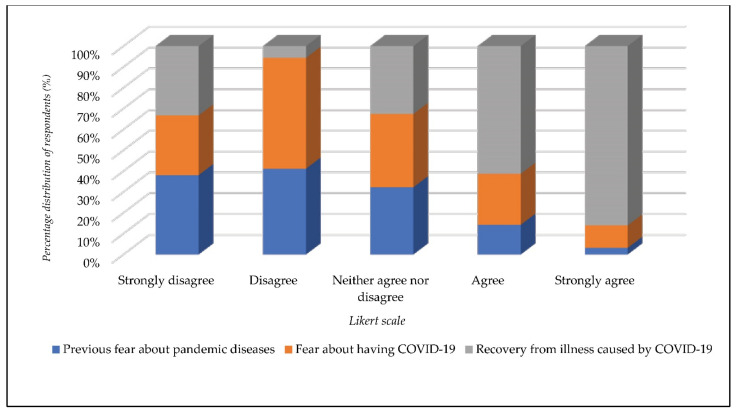
Levels of disaster fear caused by COVID-19.

The research results showed that only 3.9% of the respondents were afraid of infectious diseases before the epidemic caused by COVID-19 occurred (x = 1.71). On the other hand, 81.1% of respondents stated that they were not afraid of the epidemic caused by COVID-19 (x = 1.95). When asked to assess their chances of personal recovery after COVID-19 infection, 33% of respondents rated their recovery positively (x = 2.54) (Figure 4).

**Figure 5:**
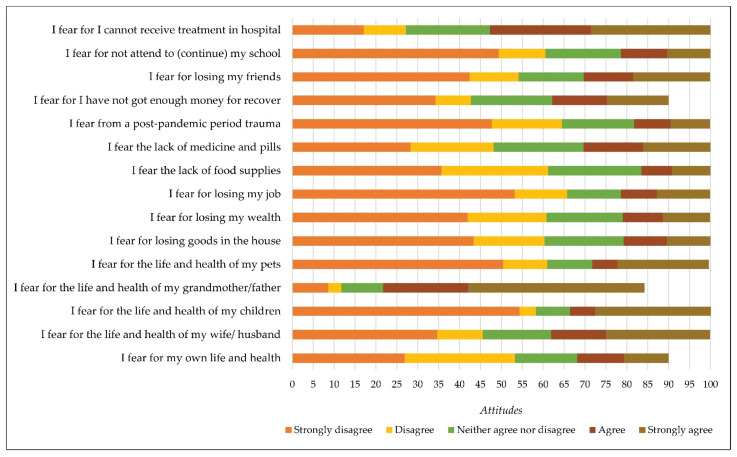
Reasons for fear caused by a coronavirus disease (COVID-19).

Subsequently, the following reasons for fear were identified: the life and health of my grandmother/father (x = 4.16; sd = 1.24), cannot receive treatment in hospital (x = 3.37; sd = 1.42), life and health of my wife/husband (x = 2.83; sd = 1.61), the lack of medicine and pills (x = 2.70; sd = 1.42), enough money for recovery (x = 2.56; sd = 1.44), own life and health (x = 2.52; sd = 1.28), losing my friends (x = 2.52; sd = 1.56), life and health of my children (x = 2.49; sd = 1.76), life and health of my pets (x = 2.39; sd = 1.64), lack of food supplies (x = 2.29; sd = 1.27), losing my wealth (x = 2.29; sd = 1.38), losing goods in the house (x =2.28; sd = 1.37), not attending (continuing) my school or education (x = 2.22; sd = 1.41), post-pandemic period trauma (x = 2.16; sd = 1.35), and losing my job (x = 2.15; sd = 1.45) (Figure 5).

**Figure 6:**
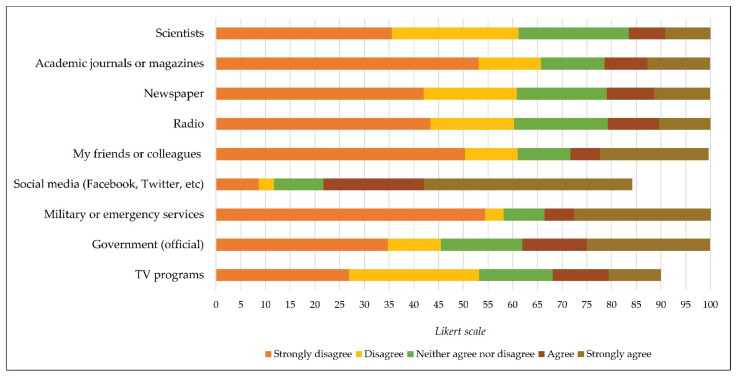
Reliable sources to acquire information about coronavirus disease (COVID-19).

In relation to the reliability of the source of information through which citizens are informed about COVID-19, the following sources were identified: scientists (x = 4.05; sd = 1.04), government (official) (x = 3.59; sd = 1.28), military or emergency services (x = 3.58; sd = 1.25), academic journals or magazines (x = 3.06; sd = 1.33), TV programs (x = 2.89; sd = 1.16), radio (x = 2.44; sd = 1.12), friends or colleagues (x = 2.37; sd = 1.02), newspaper (x = 2.18; sd = 1.06), and social media (Facebook, twitter, etc.) (x = 2.06; sd = 1.11) (Figure 6).

One-way ANOVA results showed that there was a relationship between marital status and the following variables: previous fear (*p* = 0.001), fear for my own life and health, (*p* = 0.001), fear for the life and health of my wife/husband (*p* = 0.000), fear for the life and health of my children (*p* = 0.000), fear for the life and health of my pets (*p* = 0.000), fear for losing my job (*p* = 0.005), fear the lack of medicine and pills (*p* = 0.001), and fear for losing my friends (*p* = 0.001) (Table 3). Further analyses showed that married respondents had the highest score for previous fear (x = 3.77; sd = 1.12). Single respondents had the highest score for fear caused by losing job (x = 2.13; sd = 1.46), and fear of lack of medicine and pills (x = 2.64; sd = 1.39). Divorced respondents had the highest score for fear for own life and health (x = 2.75; sd = 1.39), fear for the life and health of my wife/husband (x = 3.52; sd = 1.46), fear for the life and health of my children (x = 3.87; sd = 1.58), fear for the life and health of my pets (x = 2.53; sd = 1.69), and fear for losing my friends (x = 2.76; sd = 1.57) (Table 3).

Moreover, a statistically significant relationship was found between education level and the following variables: recovery of yourself (*p* = 0.002), fear for the life and health of my parents (*p* = 0.00), fear for the life and health of my pets (*p* = 0.006), fear for losing goods in the house (*p* = 0.001), and fear that have not got enough money for recovery (*p* = 0.002) (Table 4). Further analyses showed that respondents with primary school children had the highest score for recovery yourself (x = 2.13; sd = 1.46). Respondents with masters/doctorate degrees had the highest score for fear for their life and healthy parents (x = 4.09; sd = 1.23). Further, respondents with undergraduate degrees had the highest score for fear for the life and health of pets (x = 2.44; sd = 1.64). Respondents with secondary school education (grade 8–9) had the highest score for fear for losing goods in the house (x = 2.42; sd = 1.45) and fear of not having enough money for recovery (x = 2.37; sd = 1.46) (Table 4).

Further, a statistically significant relationship was found between age and the following variables: fear about COVID-19 (*p* = 0.003), fear for losing my job (*p* = 0.000), fear of lack of medicine and pills (*p* = 0.000), fear of post-pandemic period trauma (*p* = 0.009), fear for I have not got enough money for recovery (*p* = 0.010), fear for losing my friends (*p* = 0.003), fear for not attending to (continue) my school (*p* = 0.000) (Table 5). Younger respondents had the highest score for fear about COVID-19 (x = 1.73; sd = 0.88), fear from a post-pandemic period trauma (x = 2.50; sd = 1.41), fear that have not got enough money for recovery (x = 2.50; sd = 1.40), and fear for not attending to (continue) my school (x = 2.31; sd = 1.44). Older respondents noted the highest score for fear for losing my job (x = 2.48; sd = 1.53), fear of lack of medicine and pills (x = 3.19; sd = 1.59), and fear for losing my friends (x = 2.99; sd = 1.61) (Table 5).

One-way ANOVA results showed that there was a relationship between marital status and the variables, military or emergency services (*p* = 0.003) and government (*p* = 0.048). Married respondents had the highest score for military or emergency services (x = 3.71; sd = 1.14) and for government (x = 3.67; sd = 1.22). A statistically significant relationship (Table 6) was found between education level and the following variables: TV programs (*p* = 0.019), military or emergency services (*p* = 0.045), and academic journals or magazines (*p* = 0.001). Respondents with a secondary school education had the highest score for TV programs (x = 3.06; sd = 1.29) and military or emergency services (x = 3.70; sd = 1.26). Respondents with unfinished degrees had the highest score for academic journals or magazines (x = 3.36; sd = 1.33).

A statistically significant relationship was found between age and the variables military or emergency services (*p* = 0.000), government (*p* = 0.000), and my friends or colleagues (*p* = 0.002) (Table 6). Younger respondents had the highest score regarding military or emergency services (x = 3.70; sd = 1.21) and government (x = 3.72; sd = 1.21). Older respondents had the highest score for friends or colleagues (x = 2.67; sd = 1.82). 

### 3.3. Gender Perspective about Fear Caused by COVID-19

The results of the *t*-tests showed there were statistically significant differences between men and women in terms of previous fear (*p* = 0.000), fear about COVID-19 (*p* = 0.001), recovery of yourself (*p* = 0.008), fear for my own life and health (*p* = 0.000), fear for the life and health of my grandmother/father (*p* = 0.000), fear for losing goods in the house (*p* = 0.000), fear for the lack of medicine and pills (*p* = 0.000), fear of post-pandemic period trauma (*p* = 0.000), fear that I have not got enough money for recovery (*p* = 0.000), fear for losing my friends (*p* = 0.002), fear for not attending to (continue) my school (*p* = 0.000), fear for I cannot receive treatment in hospital (*p* = 0.000) (Table 7).

Further analyses of the obtained results showed that women, to a greater extent than men, had fears about pandemic diseases before coronavirus disease (COVID-19) occurred (x = 1.78/1.54), had fear about COVID-19 (x = 2.01/1.80), had fear for their own life and health (x = 2.64/2.25), had fear for the life and health of their grandmother/father (x = 2.64/2.25), had fear for losing goods in the house (x = 2.34/2.12), had fear for the lack of medicine and pills (x = 2.83/2.39), had fear from a post-pandemic period trauma (x = 2.34/1.73), had fear because they did not have enough money for recovery (x = 2.70/2.23), had fear for losing friends (x = 2.60/2.32), had fear for not attending to (continue) school (x = 2.32/1.97), had fear because they cannot receive treatment in hospital (x = 3.56/2.92) (Table 7).

The results of the *t*-tests showed there to be statistically significant differences in the results between men and women regarding the following information sources: TV programs (*p* = 0.000), government (official) (*p* = 0.000), military or emergency services (*p* = 0.000), social media (*p* = 0.000), radio (*p* = 0.000), newspaper (*p* = 0.005), academic journals or magazine (*p* = 0.000),and scientists (*p* = 0.000) (Table 8). Further analyses of the obtained results showed that women, to a greater extent than men, used the following information sources (Table 8): TV programs (x = 2.98/2.68), government (official) (x = 3.71/3.30), military or emergency services (x = 3.71/3.32), radio (x = 2.53/2.22), newspaper (x = 2.24/2.05), academic journals or magazines (x = 3.17/2.78), scientists (x = 3.14/2.65).

## 4. Discussion

This study examined the disaster fear caused by coronavirus disease (COVID-19) using a predictive model. The most important predictor of previous fear about pandemic diseases before coronavirus disease (COVID-19) occurred was gender, followed by marital status. Furthermore, the most important predictor for fear of COVID-19 was gender, followed by age and education level. In addition, the most important predictor for recovery opinion from pandemic COVID-19 was gender, followed by education. In sum, gender had a statistically significant effect on previous fear about pandemic diseases, fear of COVID-19 and recovery opinion from pandemic COVID-19. In addition, the impact of education on previous fear of pandemic diseases and fear of COVID-19 was not negligible. By further descriptive analyses, we found that only 3.9% of the respondents were afraid of infectious diseases before the epidemic caused by COVID-19, 81.1% of respondents stated that they were not afraid of the epidemic caused by COVID, and 33% of respondents rated their recovery positively. Examining the causes of fear, we found that the respondents were most afraid for the life and health of their wife/husband, and the least afraid of losing their job. In relation to the reliability of the source of information through which citizens were informed about COVID-19, citizens trusted scientists the most and social networks the least.

COVID-19 affects people worldwide, resulting in adverse health impacts, economic losses, death, unemployment, etc. While only 3.9% of the participants were afraid of infectious diseases before the COVID-19 pandemic, the rate was around 19% in this study. Generally, there was a statistically significant difference between men and women in terms of disaster fear. We found that women, to a greater extent than men, had a fear about pandemic diseases before coronavirus disease (COVID-19) occurred. Based on previous studies, females mention more fear across all disaster types than males [98,99,100]. Moreover, males are less inclined to express their fears than females, especially in some cultures [101]. Since females in Serbia are primarily responsible for household tasks and child care, this may make them more vulnerable to environmental risks than males. However, some previous studies have found that males [102,103] and older adults are more knowledgeable [103]. However, males may have less fear because they are more confident in their proactive activities during an emergency, and they rate their degree of self-preparedness as high [79,104,105].

According to the data in this study, before the COVID-19 pandemic, gender was a leading variable in predicting people’s fears of pandemic diseases, followed by marital status. High risk-perception toward COVID-19 was found in the community, with anxiety levels higher than in an influenza pandemic but lower than for the severe acute respiratory syndrome (SARS-2003) outbreak [106]. Studying the same issue, Bösselmann et al. [39] identified variables that predicted fear of coronavirus disease among adolescents including physical activity, age, and boredom, and then considered the association between physical activity, boredom, and quarantine experience. It was found that there was no boredom amongst physically active subjects, and they reported less fear of coronavirus disease [39]. In addition, predictors of fear included gender and the knowledge of the person diagnosed with coronavirus disease, while days spent in isolation were not a predictor of fear of the coronavirus disease, nor were they associated with feelings of loneliness [44]. Higher levels of depression, general anxiety, and insomnia were found in nurses compared with medical assistants and physicians, while physicians and health workers in COVID units showed greater coping skills. Moreover, compared to men, women reported more significant concerns about possible infection of others, anxiety, fears, depression, and post-traumatic stress [107].

Numerous studies [48,68,88,89,90,91,92] have indicated greater psychological vulnerability of members of the female population, including increased prevalence of high fear and anxiety due to the coronavirus disease pandemic. In India, where more than half of the respondents reported low levels of fear of coronavirus disease, a higher probability of levels of fear was related to gender (women), lower educational status, marital status, and healthcare status [108]. In a study conducted by Haktanir et al. [109] in a diverse Turkish group, a significantly higher level of fear was reported by members of the female population. Fear of coronavirus disease was also expressed among pregnant women in Japan, which was positively associated with health monitoring and stockpiling, while emphasis on websites and social networking sites among pregnant women was associated with less fear of coronavirus disease [110]. According to previous evidence, in people living in Bangladesh [111], fear is more present in women and the elderly. Broche-Pérez et al. [112] confirmed that gender was a significant predictor of coronavirus disease fear, and that being a woman predicted medium and high levels of fear from coronavirus disease. In addition to more pronounced fear, compared to men, women in the Cuban population reported greater concern about the reappearance of coronavirus disease and poorer self-esteem, which were related to fear reactions [113]. Among teachers, the overall prevalence of anxiety was 13.67%, while the highest was identified at 60 to 100 years in men, followed by women aged 50 to 60 years [91].

Age and COVID-19 knowledge were the other predictors for total fear scores. Graham et al. [114] found a slight positive correlation between age and stress. According to our results, fear of COVID-19 increases with age. Significantly higher levels of psychological stress were observed in health care workers who lived with the elderly and children [107].

However, a different result was found by Wang et al. [115] that age was not associated with disaster stress. Similarly, the findings of a study conducted by Haktanir et al. [109] emphasized that fear of coronavirus disease among participants in their study did not differ based on educational level, socioeconomic status, and age. Birditt et al. [116] supported a different view. In their study, older people reported less pandemic-related stress, less change in life, less social isolation, and lower negative relationship quality than younger people, while higher coronavirus disease-related stress, life change, social isolation, and negative relationship quality were reported in connection with poorer psychological wellbeing. Furthermore, worse social relationships (i.e., higher social isolation and poor quality) increased the psychological consequences of the COVID-19 pandemic (e.g., stress, life-changes) [116]. In addition to less observed stress [117] and fewer negative emotions in older adults compared to young and middle-aged adults [118], greater certainty in the information received related to coronavirus disease was found for older adults, and the perceived advantage of restrictive measures, as well as the expectation of a long time to resolve the emergency [118]. In the Brazilian population, a higher level of fear was present in residents aged 18 to 29 and in women [70]. In addition to gender and age, Nino et al. [68] found that race and ethnicity significantly shaped the perception of threat and fear of coronavirus disease.

Regarding the causes of fear related to COVID-19, gender was again the leading predictor. Age and education level were other significant predictors of COVID-19. Mangolian et al. [119] pointed to a higher intensity of fear and anxiety in girls who were single children than in other girls, and a lower level of fear and anxiety was found in girls whose mothers had a doctorate, compared to those whose mothers had a diploma degree [119]. A positive correlation was found between fear and intolerance of uncertainty and a negative correlation between the level of knowledge and fear among students, while age, gender, and type of faculty stood out as significant predictors of fear [120]. People with masters/doctorate degrees had lower anxiety than others. These results showed a decrease in fear associated with the COVID-19 pandemic, as awareness increased with education [98,121,122,123,124].

Gender and educational status were also key predictors in determining the participants’ thoughts on self-recovery from COVID-19. This study considered which factors were at the root of people’s fears about COVID-19. It was found that participants had fear mostly in relation to the following variables, “the life and health of their grandmother/father”, “cannot receive treatment in hospitals”, “life and health of their wife/husband”, “the lack of medicine and pills”, and “enough money for recovery”. Marital status was an important variable affecting fear about COVID-19.

This study had several limitations. First, considering the time sensitivity of the COVID-19 outbreak, we adopted an online survey with a convenience sampling strategy. This sampling strategy is not based on random selection; thus, the study populations are not representative of the general population. As our study does not reflect the actual pattern of the general population, our conclusion is less generalizable.

## 5. Conclusions

The world has been challenged with the novel coronavirus disease (SARS-CoV2) and its associated disease, COVID-19, since early 2020. This study provides an assessment of fear during the COVID-19 epidemic in Serbia. Our results have several significant public health implications. Females who were more educated and knowledgeable, married, and older, reported greater fear of the outbreak at various levels. Most respondents stated that they were not afraid of the epidemic caused by COVID. The most important predictor for fear of COVID-19 was gender, followed by age and education level.

The obtained research results have scientific and social implications. The scientific implications are in strengthening the empirical basis for conducting further analyses of fear during the COVID-19 epidemic and in providing a better understanding of human behavior in disasters caused by epidemics [125,126,127]. The study also complements the scientific fund of knowledge in disaster studies and addresses shortcomings in terms of understanding the impact of demographic and socio-economic factors on people’s fears during disasters caused by epidemics. 

From a societal perspective, the research results enable decision-makers to scientifically create and implement strategies, programs, and campaigns for the psycho-social support of people in disasters caused by epidemics. In addition, understanding the impact on people’s fear of disasters caused by epidemics allows the design and adaptation of strategic, tactical, and operational interventions for disaster risk communications and the implementation of public mental health policies.

More research should be carried out on fear according to ethnicity, religion, and geographical differences. First, studies can be conducted on the duration of disasters and the psychological effects of direct experience. Second, the relationship between the economic situation and participants’ emotional reactions to the pandemic could be investigated. Third, the impact of possible disasters on individuals living in urban and rural areas could be compared. Our findings directly inform understanding of perceived fear during the COVID-19 outbreak. Fourth, the inability to conduct a longitudinal study can be characterized as a deficiency due to uncertainty around the start and end of the pandemic. In order to discern the psychological changes in pandemic disasters, longitudinal examination of individuals during COVID-19 or another epidemic disease process will allow useful data to be obtained.

## Figures and Tables

**Table 1:** Basic socioeconomic and demographic information of respondents (*n* = 1226).

Variable	Category	(f)	%
Male	Male	580	47.3
	Female	646	52.6
Age	18–30	650	53.02
	31–45	380	31
	46–64	196	15.9
Marital status	Single	491	40
	Married	512	41.8
	Divorced	212	17.3
	Widower/Widow	11	0.9
Education	Primary Sch. (grade 4–5)	89	7.26
	Secondary Sch. (grade 8–9)	690	56.2
	High school (grade 11–12)	85	6.9
	Undergraduate	272	22.19
	Master/doctorate	90	7.34
Number of children	1	570	46.49
	2–3	111	9.05
	4+	65	5.30
	None (0)	480	39.15
COVİD-19 knowledge	Very poor	34	2.8
	Poor	91	7.4
	Moderate	529	43.1
	Good	470	38.3
	Excellent	102	8.3
TOTAL		1226	100

**Table 2:** Results of multivariate regression analysis concerning fear for coronavirus disease (*n* = 1226).

Predictor Variable	Previous Fear			Fear About COVID-19			Recover of Yourself		
	B	SE	β	B	SE	β	B	SE	β
Gender	−0.234	0.056	−0.119 **	−0.218	0.063	−0.099 **	0.261	0.101	0.074 *
Age	0.036	0.073	0.018	−0.223	0.082	−0.098 *	−0.215	0.132	−0.059
Marital status	−0.155	0.056	−0.085 *	−0.098	0.063	−0.048	0.017	0.101	0.005
Education level	−0.105	0.370	−0.008	−1.02	0.414	−0.071 *	1.704	0.668	0.073 *
Children	−0.007	0.087	−0.003	−0.164	0.098	−0.061	−0.049	0.158	−0.011
Knowledge of coronavirus	−0.009	0.085	−0.003	0.054	0.095	0.016	0.246	0.153	0.046
Adjusted R^2^	0.16			0.18			0.12		

* *p* ≤ 0.05; ** *p* ≤ 0.01; B: unstandardized (B) coefficient; SE: std. error; β: standardized (β) coefficients. Note: males, young (<30), single-headed households, secondary-school, respondents with one child, and people with very poor and poor knowledge have been coded as 0; 1 has been assigned otherwise (females, middle-aged and old, married, undergraduate, respondents with two or more children; people with good and excellent knowledge).

**Table 3:** One-way ANOVA results of the relations between the variables about fear caused by COVID-19 and marital status.

Variable	*F*	*p*	Sum of Squares	Mean Square	Single	Married	Divorced	Widow
					M (SD)	M (SD)	M (SD)	M (SD)
Previous fear	5.43	0.001 **	13.02	4.34	1.62 (0.92)	3.77 (1.12)	2.68 (1.05)	2.45 (1.03)
Fear about COVID-19	1.54	0.200	4.70	1.57	1.88 (0.99)	2.01 (1.00)	1.96 (1.05)	2.18 (0.87)
Recovery of yourself	1.12	0.339	8.82	2.94	2.52 (1.65)	2.49 (1.60)	2.68 (1.59)	3.09 (1.04)
I fear for my own life and health	5.48	0.001 **	26.87	8.95	2.38 (1.26)	2.55 (1.24)	2.75 (1.39)	2.18 (1.07)
I fear for the life and health of my wife/husband	52.49	0.000 **	363.08	121.02	2.18 (1.61)	3.16 (1.44)	3.52 (1.46)	2.91 (1.30)
I fear for the life and health of my children	66.33	0.000 **	535.30	178.43	2.11 (1.63)	2.24 (1.66)	3.87 (1.58)	3.09 (1.57)
I fear for the life and health of my parents	2.16	0.090	10.04	3.34	4.13 (1.26)	4.25 (1.18)	4.01 (1.34)	4.45 (0.68)
I fear for the life and health of my pets	4.31	0.005 *	34.49	11.49	2.24 (1.58)	2.29 (1.59)	2.53 (1.69)	2.45 (1.50)
I fear for losing goods in the house	0.44	0.718	2.56	0.85	2.22 (1.34)	2.31 (1.37)	2.31 (1.48)	2.27 (0.90)
I fear losing my wealth	1.99	0.113	11.46	3.82	2.20 (1.32)	2.32 (1.40)	2.46 (1.49)	2.00 (1.09)
I fear for losing my job	2.71	0.044 *	17.27	5.75	2.13 (1.46)	2.08 (1.42)	2.06 (1.51)	2.11 (1.32)
I fear the lack of food supplies	1.91	0.125	9.29	3.09	2.23 (1.19)	2.27 (1.29)	2.46 (1.38)	2.64 (0.94)
I fear the lack of medicine and pills	5.65	0.001 **	33.98	11.32	2.64 (1.39)	2.61 (1.38)	2.01 (1.37)	2.55 (1.46)
I fear from a post-pandemic period trauma	1.81	0.142	9.97	3.32	2.08 (1.29)	2.15 (1.35)	2.32 (1.46)	2.55 (1.57)
I fear for I have not got enough money for recovery	1.74	0.156	10.86	3.62	2.47 (1.44)	2.57 (1.40)	2.73 (1.53)	2.73 (1.19)
I fear for losing my friends	5.77	0.001 **	41.79	13.93	2.49 (1.59)	2.42 (1.50)	2.76 (1.58)	2.66 (1.47)
I fear for not attend to (continue) my school	0.89	0.444	5.35	1.78	2.26 (1.38)	2.23 (1.45)	2.08 (1.36)	2.18 (1.40)
I fear for I cannot receive treatment in the hospital	0.96	0.407	5.91	1.97	3.30 (1.45)	3.41 (1.37)	3.42 (1.50)	3.82 (1.32)

* *p* ≤ 0.05; ** *p* ≤ 0.01.

**Table 4:** One-way ANOVA results of the relations between the variables about fear caused by COVID-19 and education level.

Variable	*F*	*p*	Sum of Squares	Mean Square	Primary Sch. (Grade 4–5)	Secondary Sch. (Grade 8–9)	High School (Grade 11–12)	Undergraduate	Master/Doctorate
					M (SD)	M (SD)	M (SD)	M (SD)	M (SD)
Previous fear	0.519	0.669	1.25	0.42	1.67 (1.63)	1.65 (0.90)	1.72 (0.90)	1.75 (0.82)	1.64 (0.89)
Fear about COVID-19	2.54	0.055	7.71	2.57	1.00 (0.00)	1.88 (0.96)	1.98 (1.02)	1.99 (1.00)	1.87 (0.99)
Recovery of yourself	5.04	0.002 *	39.30	13.10	2.13 (1.46)	1.85 (1.35)	2.01 (1.34)	2.5 (1.31)	1.89 (1.40)
I fear for my own life and health	1.41	0.236	7.02	2.34	4. 33 (1.63)	2.34 (1.58)	2.56 (1.63)	2.49 (1.61)	2.76 (1.56)
I fear for the life and health of my wife/husband	0.10	0.959	0.79	0.26	2.00 (1.67)	2.51 (1.35)	2.57 (1.28)	2.53 (1.39)	2.36 (1.08)
I fear for the life and health of my children	2.27	0.079	21.20	7.06	3.00 (2.19)	2.79 (1.66)	2.84 (1.61)	2.82 (1.41)	2.85 (1.46)
I fear for the life and health of my parents	6.65	0.000 **	30.51	10.17	3.85 (1.25)	3.90 (1.28)	4.05 (1.35)	3.95 (1.18)	4.09 (1.23)
I fear for the life and health of my pets	4.12	0.006 *	33.07	11.01	2.17 (1.58)	2.30 (1.62)	2.21 (1.68)	2.44 (1.64)	1.99 (1.47)
I fear for losing goods in the house	5.88	0.001 **	33.21	11.07	2.21 (1.37)	2.42 (1.45)	2.34 (1.64)	2.15 (1.58)	1.98 (1.27)
I fear losing my wealth	0.93	0.423	5.93	1.79	1.67 (1.63)	2.42 (1.45)	2.30 (1.35)	1.65 (0.95)	1.87 (1.26)
I fear for losing my job	2.49	0.058	15.89	5.29	1.66 (1.63)	2.37 (1.46)	2.29 (1.37)	2.40 (1.57)	2.20 (1.30)
I fear the lack of food supplies	3.05	0.028	14.73	4.91	1.67 (1.63)	2.28 (1.55)	2.16 (1.45)	1.90 (1.26)	2.00 (1.38)
I fear the lack of medicine and pills	1.30	0.272	7.92	2.64	1.65 (1.63)	2.41 (1.39)	2.29 (1.22)	2.06 (1.21)	1.85 (1.95)
I fear from a post-pandemic period trauma	3.49	0.015	19.13	6.37	3.33 (1.96)	2.68 (1.49)	2.73 (1.40)	2.53 (1.32)	2.15 (1.58)
I fear for I have not got enough money for recovery	5.11	0.002 *	31.57	10.52	2.18 (1.36)	2.37 (1.46)	2.10 (1.48)	2.05 (1.39)	1.98 (1.40)
I fear for losing my friends	2.22	0.084	16.23	5.41	2.00 (1.67)	2.13 (1.33)	2.23 (1.38)	1.84 (1.17)	1.54 (1.58)
I fear for not attend to (continue) my school	9.83	0.000 **	57.70	19.23	3.50 (1.97)	2.24 (1.45)	2.30 (1.42)	1.69 (1.06)	3.01 (1.01)
I fear for I cannot receive treatment in the hospital	1.62	0.059	15.94	5.31	3.67 (2.06)	3.27 (1.47)	3.45 (1.39)	3.15 (1.46)	3.01 (1.95)

* *p* ≤ 0.05; ** *p* ≤ 0.01.

**Table 5:** One-way ANOVA results of the relations between the variables about fear caused by COVID-19 and age.

Variable	*F*	*p*	Sum of Squares	Mean Square	18–30	31–45	46–64
					M (SD)	M (SD)	M (SD)
Previous fear	1.30	0.272	3.15	1.05	1.91 (0.98)	2.17 (1.09)	1.83 (0.96)
Fear about COVID-19	4.71	0.003 *	14.23	4.76	1.73 (0.88)	1.69 (0.87)	1.71 (0.85)
Recovery of yourself	1.75	0.155	13.70	4.57	2.48 (1.63)	2.71 (1.61)	2.58 (1.47)
I fear for my own life and health	1.42	0.234	7.05	2.35	2.51 (1.29)	2.52 (1.18)	2.69 (1.32)
I fear for the life and health of my wife/husband	9.36	0.000 **	71.44	23.81	2.70 (1.62)	3.06 (1.55)	3.42 (1.45)
I fear for the life and health of my children	41.10	0.000 **	349.71	116.57	2.20 (1.66)	2.90 (1.80)	3.94 (1.58)
I fear for the life and health of my parents	7.26	0.000 **	32.94	10.98	4.25 (1.16)	3.95 (1.36)	3.98 (1.41)
I fear for the life and health of my pets	1.91	0.053	23.30	7.76	2.45 (1.67)	2.11 (1.50)	2.45 (1.56)
I fear for losing goods in the house	1.85	0.056	16.20	5.40	2.96 (1.35)	2.18 (1.40)	2.48 (1.52)
I fear losing my wealth	1.19	0.310	6.88	2.29	2.27 (1.36)	2.34 (1.39)	2.48 (1.55)
I fear for losing my job	11.44	0.000 **	71.32	23.77	2.02 (1.41)	2.37 (1.51)	2.48 (1.53)
I fear the lack of food supplies	1.72	0.160	8.35	2.78	2.26 (1.24)	2.37 (1.27)	2.48 (1.49)
I fear the lack of medicine and pills	7.14	0.000 **	42.70	14.23	2.63 (1.41)	2.78 (1.31)	3.19 (1.59)
I fear from a post-pandemic period trauma	3.88	0.009 *	21.24	7.08	2.50 (1.41)	2.14 (1.44)	2.13 (1.32)
I fear for I have not got enough money for recovery	3.79	0.010 *	23.54	7.84	2.50 (1.40)	2.41 (1.50)	2.38 (1.48)
I fear for losing my friends	4.67	0.003 *	33.85	11.28	2.44 (1.65)	2.59 (1.53)	2.99 (1.61)
I fear for not attend to (continue) my school	8.48	0.000 **	49.71	16.57	2.31 (1.44)	1.80 (1.19)	2.27 (1.38)
I fear for I cannot receive treatment in the hospital	0.55	0.645	3.38	1.13	3.35 (1.41)	3.44 (1.40)	3.40 (1.52)

* *p* ≤ 0.05; ** *p* ≤ 0.01.

**Table 6:** One-way ANOVA results comparing sources citizens use to receive information about COVID-19 by marital status, education level, and age.

Variable	Marital Status				Education Level				Age			
	*F*	*p*	Sum of Squares	Mean Square	*F*	*p*	Sum of Squares	Mean Square	*F*	*p*	Sum of Squares	Mean Square
TV programs	0.960	0.411	3.92	1.30	3.32	0.019 *	13.50	4.50	1.98	0.114	8.05	2.68
Government (official)	2.64	0.048 *	13.03	4.34	1.76	0.153	8.69	2.90	14.01	0.000 **	67.17	22.39
Military or emergency services	4.78	0.003 *	22.63	7.54	2.68	0.045 *	12.76	4.25	11.54	0.000 **	53.75	17.91
Social media (Facebook, Twitter)	0.219	0.883	0.81	0.27	0.455	0.714	1.68	0.56	0.227	0.878	0.84	0.28
My friends or colleagues	1.53	0.203	4.80	1.60	2.07	0.102	6.46	2.15	4.97	0.002 *	15.25	5.08
Radio	2.29	0.077	8.69	2.89	1.52	0.207	5.78	1.92	1.88	0.131	7.09	2.36
Newspaper	2.82	0.037	9.63	3.21	1.84	0.057	9.68	3.22	1.67	0.171	5.66	1.88
Academic journals or magazines	4.29	0.005	22.60	7.53	5.50	0.001 **	28.90	9.63	0.963	0.409	5.10	1.70
Scientists	1.20	0.306	3.92	1.30	0.815	0.485	2.65	0.88	1.63	0.179	5.32	1.77

* *p* ≤ 0.05; ** *p* ≤ 0.01.

**Table 7:** Independent samples *t*-test results for gender and variables relating to fear caused by COVID-19.

Variable	Gender					
	*F*	*t*	Sig. (2-Tailed)	df	Male X (SD)	Female X (SD)
Previous fear	14.98	−4.58	0.000 **	804	1.54 (0.768)	1.78 (0.933)
Fear about COVID-19	0.000	−3.31	0.001 **	1224	1.80 (0.941)	2.01 (1.02)
Recovery of yourself	2.66	2.67	0.008 *	1224	2.73 (1.67)	2.46 (1.58)
I fear for my own life and health	1.81	−4.95	0.000 **	1224	2.25 (1.23)	2.64 (1.28)
I fear for the life and health of my wife/husband	0.440	−1.10	0.270	1224	2.75 (1.58)	2.86 (1.62)
I fear for the life and health of my children	10.49	−1.04	0.285	1224	2.41 (1.69)	2.52 (1.79)
I fear for the life and health of my parents	3.64	−3.84	0.000 **	1224	3.95 (1.31)	4.25 (1.20)
I fear for the life and health of my pets	2.18	−1.60	0.109	1224	2.27 (1.61)	2.44 (1.64)
I fear for losing goods in the house	0.350	−2.65	0.008 *	1224	2.12 (1.39)	2.34 (1.36)
I fear for losing my wealth	0.412	−1.27	0.204	1224	2.22 (1.41)	2.33 (1.33)
I fear losing my job	5.19	−1.56	0.118	728	2.05 (1.39)	2.19 (1.48)
I fear the lack of food supplies	3.23	−1.44	0.159	1224	2.21 (1.22)	2.32 (1.29)
I fear the lack of medicine and pills	0.132	−4.95	0.000 **	851	2.39 (1.39)	2.83 (1.41)
I fear from a post-pandemic period trauma	62.40	−7.99	0.000 **	746	1.73 (1.11)	2.34 (1.40)
I fear for have not got enough money to recover	12.18	−5.42	0.000 **	772	2.23 (1.33)	2.70 (1.46)
I fear for losing my friends	27.50	−3.03	0.002 **	736	2.32 (1.41)	2.60 (1.61)
I fear for not attending to (continue) my school	12.47	−4.18	0.000 **	634	1.97 (1.32)	2.32 (1.43)
I fear for I cannot receive treatment in the hospital	5.59	−7.04	0.000 **	1224	2.92 (1.47)	3.56 (1.36)

* *p* ≤ 0.05; ** *p* ≤ 0.01.

**Table 8:** Independent samples *t*-test results for gender and the information sources of citizens to receive information about COVID-19.

Variable	Gender					
	*F*	Sig. (2-Tailed)	*t*	df	Male X (SD)	Female X (SD)
TV programs	17.36	0.000 **	−4.15	1224	2.68 (1.24)	2.98 (1.11)
Government (official)	8.17	0.000 **	−5.22	1224	3.30 (1.35)	3.71 (1.23)
Military or emergency services	10.42	0.000 **	−4.81	625	3.32 (1.34)	3.71 (1.20)
Social media	22.72	0.017 *	2.38	612	2.18 (1.20)	2.00 (1.06)
My friends or colleagues	4.08	0.053	1.71	1224	2.44 (1.06)	2.33 (0.999)
Radio	0.306	0.000 **	−4.47	1224	2.22 (1.10)	2.53 (1.12)
Newspaper	0.315	0.005 *	−2.79	1224	2.05 (1.10)	2.24 (1.04)
Academic journals or magazines	5.02	0.000 **	−4.72	1224	2.78 (1.37)	3.17 (1.29)
Scientists	3.19	0.000 **	−3.50	1224	3.89 (1.10)	4.12 (1.00)

* *p* ≤ 0.05; ** *p* ≤ 0.01.

## Appendix A

Dear citizens, today, coronavirus disease (COVID-19) is a new and pandemic illness globally. It creates an incredible amount of anxiety and stress on people who have experienced or are not around it. Almost all people are talking about it, and countries have taken precautions to prevent coronavirus disease damages. WHO declared coronavirus disease (COVID-19) as a pandemic. We are carrying out a study to determine what the means of COVID-19 fears have for you in your life. We are interested in what kind of anxiety or fear is related to coronavirus disease as well. Remember that your participation in the survey is anonymous, and your answers are confidential: your answers won’t be treated individually but as an inseparable part of the overall research data. Your answers are most important for this study. Please answer all these questions freely.

1. Country: ……………………….
2. Gender: (a) Male   (b) Female   (c) …………….
3. Age: ……………………….
4. Marital status:
  (a) single   (b) married   (c) divorced   (d) widow
5. Education level:
  (a) Primary school   (b) Secondary school   (c) High school   (d) Undergraduate   (e) Master/doctorate
6. The number of children:
  (a) any   (b) 1   (c) 2–3   (d) 4+
7. What do you think about your coronavirus knowledge (please mark with X):
  (a) very poor   (b) poor   (c) moderate   (d) good   (e) excellent
8. Please mark from 1 to 5 for the level of your fear.
  	**Never**	**Rarely**	**Sometimes**	**Often**	**Always**
  	**1**	**2**	**3**	**4**	**5**
  1. Have you ever fear about pandemic diseases until coronavirus disease (COVID-19) occurred?					
  1. Do you have any fear about having coronavirus disease (COVID-19) disease?					
  1. If you have coronavirus disease illness, what do you think about a recovery yourself?					
9. What is the reason of fear caused by coronavirus disease (COVID-19)? Please mark from 1 to 5 for the level of your fear itemized (mark with X).
  	**Strongly** **Disagree**	**Disagree**	**Neither Agree nor Disagree**	**Agree**	**Strongly Agree**
  	**1**	**2**	**3**	**4**	**5**
  I fear for my own life and health.					
  I fear for the life and health of my wife/husband.					
  I fear for the life and health of my children.					
  I fear for the life and health of my grandmother/father.					
  I fear for the life and health of my pets.					
  I fear for losing goods in the house.					
  I fear for losing my wealth (money, house, etc).					
  I fear for losing my job.					
  I fear the lack of food supplies.					
  I fear the lack of medicine and pills.					
  I fear from a post-pandemic period trauma.					
  I fear that I have not got enough money for recovery.					
  I fear losing my friends.					
  I fear for not attend to (continue) my school.					
  I fear for I cannot receive treatment in hospital.					
  Other ………………………… (please write)					
10. Which sources are more reliable to get information about coronavirus disease (COVID-19)? (mark with x).
  	**Strongly** **Disagree**	**Disagree**	**Neither Agree nor Disagree**	**Agree**	**Strongly Agree**
  	**1**	**2**	**3**	**4**	**5**
  (a) TV programs					
  (b) Government (official)					
  (c) Military or emergency services					
  (d) Social media (Facebook, twitter, etc)					
  (e) My friends or colleagues					
  (f) Radio					
  (g) Newspaper					
  (h) Academic journals or magazine					
  (i) Scientists					
  Other ……………………………………………. (please write)					
